# DNA methylation loci identification for pan-cancer early-stage diagnosis and prognosis using a new distributed parallel partial least squares method

**DOI:** 10.3389/fgene.2022.940214

**Published:** 2022-10-19

**Authors:** Qi-en He, Jun-xuan Zhu, Li-yan Wang, En-ci Ding, Kai Song

**Affiliations:** ^1^ School of Chemical Engineering and Technology, Tianjin University, Tianjin, China; ^2^ Tianjin First Central Hospital, Tianjin, China

**Keywords:** DNA methylation, partial least squares, MapReduce, pan-cancer analysis, early-stage tumor diagnosis and prognosis

## Abstract

Aberrant methylation is one of the early detectable events in many tumors, which is very promising for pan-cancer early-stage diagnosis and prognosis. To efficiently analyze the big pan-cancer methylation data and to overcome the co-methylation phenomenon, a MapReduce-based distributed and parallel-designed partial least squares approach was proposed. The large-scale high-dimensional methylation data were first decomposed into distributed blocks according to their genome locations. A distributed and parallel data processing strategy was proposed based on the framework of MapReduce, and then latent variables were further extracted for each distributed block. A set of pan-cancer signatures through a differential co-expression network followed by statistical tests was further identified based on their gene expression profiles. In total, 15 TCGA and 3 GEO datasets were used as the training and testing data, respectively, to verify our method. As a result, 22,000 potential methylation loci were selected as highly related loci with early-stage pan-cancer diagnosis. Of these, 67 methylation loci were further identified as pan-cancer signatures considering their gene expression as well. The survival analysis as well as pathway enrichment analysis on them shows that not only these loci may serve as potential drug targets, but also the proposed method may serve as a uniform framework for signature identification with big data.

## Introduction

Early diagnosis of cancer has been a worldwide hotspot of research because it can obviously increase the opportunities for effective treatment and appropriate monitoring of cancer patients. Since it has been well-known that distinct types of human cancer share similar traits, including rapid cell proliferation, the ability to migrate, and seed malignant tumors in distal locations (Zhang et al., 2012), tremendous efforts have been taken in the development of reliable and cost-effective early detection methods for common cancers (Irizarry et al., 2009). It has been shown that the aberrant variation of methylation, which is a major pattern of epigenetics, is an early event in many tumors. It may also be one of the first detectable biomarkers for the early detection of cancer (Xu et al., 2017; Luo et al., 2020). Correspondingly, the methylation pan-cancer study is an emerging research hotspot, which is dedicated to find common or cancer-specific diagnostic or prognostic biomarkers from a variety of cancers (Yang et al., 2017; Ding et al., 2019; Tian et al., 2019).

Although good outcomes have been achieved over these studies, there are still improvements that need to be made: 1) there are few pan-cancer methylation studies focusing on the early diagnosis of cancer. 2) Most of the existing methods do not consider the functional relationships between methylation loci or even the co-methylation phenomenon, resulting in many false-positive results.

It is now a well-recognized fact that the main difference between cancer and normal cells is a complex landscape of genetic and epigenetic aberrations, which usually cause a rewiring of gene regulatory networks (GRNs) at a system level, finally impairing normal cell physiology (Hanahan and Weinberg, 2011). Correspondingly, signature methylation loci responsible for pan-cancer early-stage diagnosis should be functionally related to each other by regulating their own expression levels first (Matys et al., 2003). Therefore, our study is aimed to identify functionally related methylation loci for pan-cancer early diagnosis. It inevitably evolves the analysis of pan-cancer genome-wide methylation and expression data.

Unfortunately, the most widely used methylation platform is the Infinium HumanMethylation450 or 850 k platform. It includes 485,000 or 850,000 CpG loci covering more than 99% of RefSeq genes (Price et al., 2013). Such high-dimensional variables but only thousands of pan-cancer patients bring up a typical problem: big data with a comparatively very small sample size.

A new wave of deep learning in both academic and industrial fields has gradually developed deep ANN (Elmarakeby et al., 2021), graph convolutional networks (Wang et al., 2021), and other deep learning methods with multilayer nonlinear structures due to their superior visualization and classification performance. Unfortunately, for machine learning methods, especially for deep learning methods, the smaller the training sample size is, the robustness or generalization ability of the trained model is less. Additionally, although DeepLIFT (Trevino et al., 2021) and other interpretation methods have been available for deep learning methods, it is still hard for them to identify biomarkers. In short, both high-dimensional big data and the biomarker identification of our study exclude deep learning methods.

More importantly, it has been found that closer neighboring CpG sites are more likely to share the same methylation status (Affinito et al., 2020). An effective way to overcome this co-methylation situation is to divide the methylation loci into different groups and then select the important ones from the most important ones of each individual group.

**FIGURE 1:**
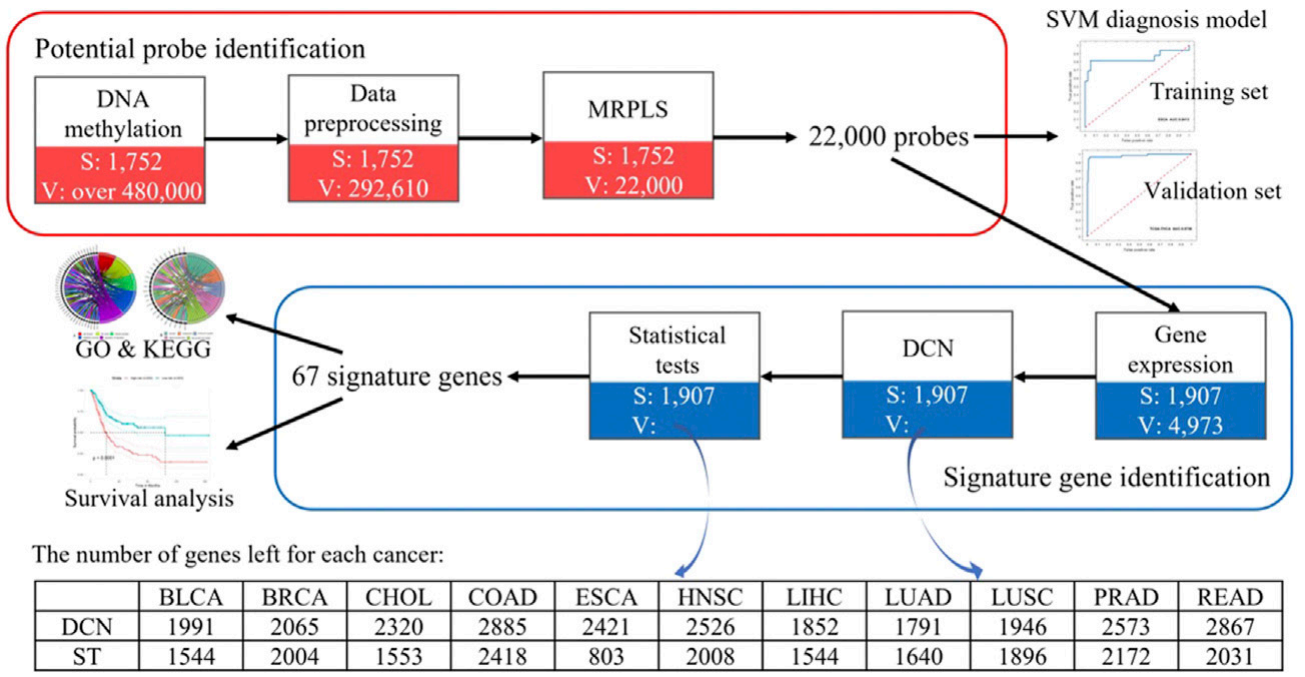
Overall pipeline of identified methylation loci for early-stage pan-cancer diagnosis. MRPLS, MapReduce-based partial least squares; DCN, differential co-expression network; ST, statistical test; S, the number of samples; V, the number of variables.

Therefore, to speed up the analysis, to avoid false-positive results caused by co-methylation, and to improve the performance for such a big-data problem, a MapReduce-based partial least squares (MRPLS) method was proposed. The overall pipeline of this study is shown in Figure 1.

## Materials and methods

### Datasets

Level 3 DNA methylation (HumanMethylation450), level-3 RNA-Seq V2, and clinical data were downloaded from TCGA. Among them, the data of 12 types of cancer were used as the training dataset. The data of other three types of cancer from TCGA and three GEO datasets (GSE54503, GSE63409, and GSE66695) were collected as the independent test dataset. Their details are summarized in Table 1.

**TABLE 1:** Details of the training and independent test sets.

Training set						
Cancer	Full name/access number	DM^a^		GE^b^		Stage
		T^c^	N^d^	T	N	
BLCA	Bladder urothelial carcinoma	52	21	132	18	I and II
BRCA	Breast invasive carcinoma	308	96	176	113	I
CHOL	Cholangiocarcinoma	28	9	19	9	I and II
COAD	Colon adenocarcinoma	43	38	78	41	I
ESCA	Esophageal carcinoma	39	16	16	11	I
HNSC	Head and neck squamous cell carcinoma	36	50	94	44	I
LIHC	Liver hepatocellular carcinoma	263	50	172	50	I and II
LUAD	Lung adenocarcinoma	248	32	282	59	I
LUSC	Lung squamous cell carcinoma	143	42	242	49	I
PAAD	Pancreatic adenocarcinoma	21	10	21	4	I
PRAD	Prostate adenocarcinoma	140	50	187	52	I and II
READ	Rectum adenocarcinoma	10	7	28	10	I
	Total	1,331	421	1,447	460	
Independent test set						
KIRC	Kidney renal clear cell carcinoma	155	160	—	—	I
KIRP	Kidney renal papillary cell carcinoma	167	45	—	—	I
THCA	Thyroid carcinoma	285	56	—	—	I
Liver	GEO-GSE54503	66	66	—	—	—
AML^e^	GEO-GSE63409	44	30	—	—	—
Breast	GEO-GSE66695	80	40	—	—	—

^a^ DM, DNA methylation.

^b^ GE, gene expression.

^c^ T, tumor samples.

^d^ N, non-malignant samples.

^e^ AML, acute myelogenous leukemia.

The methylation level of each locus is represented as a beta-value *β*, which is defined as the ratio of the methylated allele intensity and the overall intensity (Bibikova et al., 2011):

$$\beta =\frac{M}{M+U+100},$$ (1)

where *M* is the methylated intensity and *U* is the unmethylated intensity of each locus. *β* is a continuous variable with a value between 0 and 1, where 0 means no methylation and 1 means completely methylated.

### Data preprocessing

For each type of cancer, there are over 485,000 loci in the downloaded methylation data with many missing values represented as “NA.” This is due to the masking of CpGs owing to single-nucleotide polymorphisms (SNPs) with a high minor allele frequency within 10 bp of the targeted CpGs or a substantial overlap between probe sequences and repetitive elements (Yang et al., 2017). Therefore, the following multi-step preprocessing procedure was performed before any further analysis to reduce the computational complexity as well as to improve the accuracy of the final results:

1) Identifying common loci among all types of cancers.
2) Removing the explicitly built-in SNP loci (identifiers start with “rs”) and non-CpG-targeting loci (identifiers start with “ch”).
3) Removing loci with “NA” values in more than 30% of the samples.
4) Replacing “NA” values with the corresponding average values of non-NA values of other samples.
5) Removing loci with SD (standard deviation) < 0.01 to reduce significantly unrelated or redundant loci.
6) An empirical Bayesian method ComBat (Johnson et al., 2007) is employed to eliminate batch effects caused by the system bench effect or abiotic differences using R package “sva.”

At this point, 292,610 high-quality loci are obtained for each type of cancer.

### The new MapReduce-based partial least squares method

Several analysis methods like SVM (support vector machine) were used in our study. For readers’ convenience and for the clear context, only PLS and MapReduce, which are the basis for our newly proposed MRPLS, are briefly introduced here. Other regular existing methods are available in the Supplementary Material.

⁃ Partial least squares (PLS)

PLS is a widely used algorithm for modeling relationships between sets of observed variables. Although PLS was not originally designed as a tool for statistical discrimination, applied scientists routinely use PLS for classification, and there is substantial empirical evidence to suggest that it performs well in that role (Barker and Rayens, 2003). It iteratively extracts the latent variables (LVs) *t*_i_, *u*_*i*_, X-loading vectors *p*_*i*_, and Y-weight vectors *q*_*i*_ from *X* and *Y* matrices in decreasing order of their corresponding singular values as follows (Word et al., 2001):

$$X=\overset{A}{\underset{i=1}{\sum }}t_{i}p_{i}^{T}+E=TP^{T}+E,$$ (2)

$$Y=\overset{A}{\underset{i=1}{\sum }}u_{i}q_{i}^{T}+F=UQ^{T}+F,$$ (3)

where E and F are the residual matrices of X and Y, respectively; n is the number of variables; and i=1,2,⋯,A, where A (A << n) is the number of LVs, which is usually determined by cross-validation. The non-linear iterative partial least squares (NIPALS) method is the most widely used algorithm for PLS (Mehmood et al., 2012), and the details of the NIPALS method is available in the Supplementary Material.

The variable importance in projection (VIP) index based on PLS can be used to evaluate the importance of each variable (in our case, it is the methylation value of each locus) in the classification model (Mehmood et al., 2012). The definition is

$$VIP=\sqrt{n\times \left(k/sum\left(s\right)\right)},$$ (4)

$$s=diag\left(T^{\prime }\times T\times Q\times Q^{\prime }\right),$$ (5)

$$k=s^{\prime }\times w,$$ (6)

where k stands for the explained variance of Y by each variable, s represents the total variance explained by LVs, and w is the unitized form of W.

⁃ MapReduce framework

MapReduce is a programming framework proposed by Google for modeling and analyzing those massive amounts of data in a parallel manner. The MapReduce programming framework employs the Hadoop Distributed File System (HDFS) to store data. It saves researchers from organizing and managing files in computer stations. Researchers only need to pay attention on how the Map and Reduce functions are written. In the MapReduce model, all the computation is organized by <key, value> pairs, especially in the Map phase, each worker node takes the initial organized <key1, value1> pairs as input and produces a list of intermediate <key2, value2> pairs as output. This can be represented as

Map:<key1,value1>→list<key2,value2>. (7)

Then, the system merges and groups these intermediate pairs by the same key2 and passes them to the Reduce function. Afterward, the Reduce function takes a key and a related value list as input and generates the expected < key2, value3> pair lists as output, which can be represented as

Reduce:<key2,list(value2)>→list<key2,value3>. (8)

**FIGURE 2:**
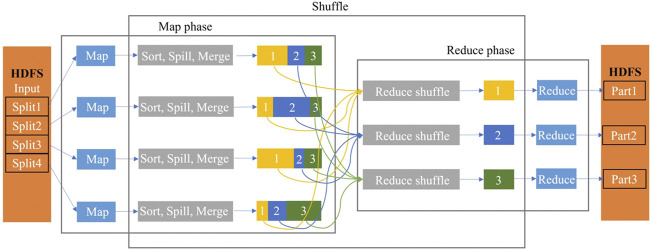
Data flow overview of the MapReduce model with four Map workers and three Reduce workers.

The simplified data flow overview of MapReduce is shown in Figure 2.

### The newly proposed MRPLS method

In the traditional PLS method, VIP is usually used to evaluate the importance of features in the classification model (Mehmood et al., 2012). When it comes to VIP calculation for big data, however, NIPALS would become very slow or even out of the computer’s memory. Hence, MRPLS (MapReduce-based partial least squares) algorithm was first proposed by us to handle massive amounts of biological data. As mentioned previously, the core task of MRPLS is to design appropriate <key, value> pairs for the Map and Reduce process, respectively. Therefore, MRPLS is designed consisting of three MapReduce modules in series:

♦ MapReduce1 is used to calculate w;
♦ MapReduce2 takes w and X as input and calculates t and p, respectively;
♦ MapReduce3 takes t, p, and X as input to calculate new X for the next iteration.

**FIGURE 3:**
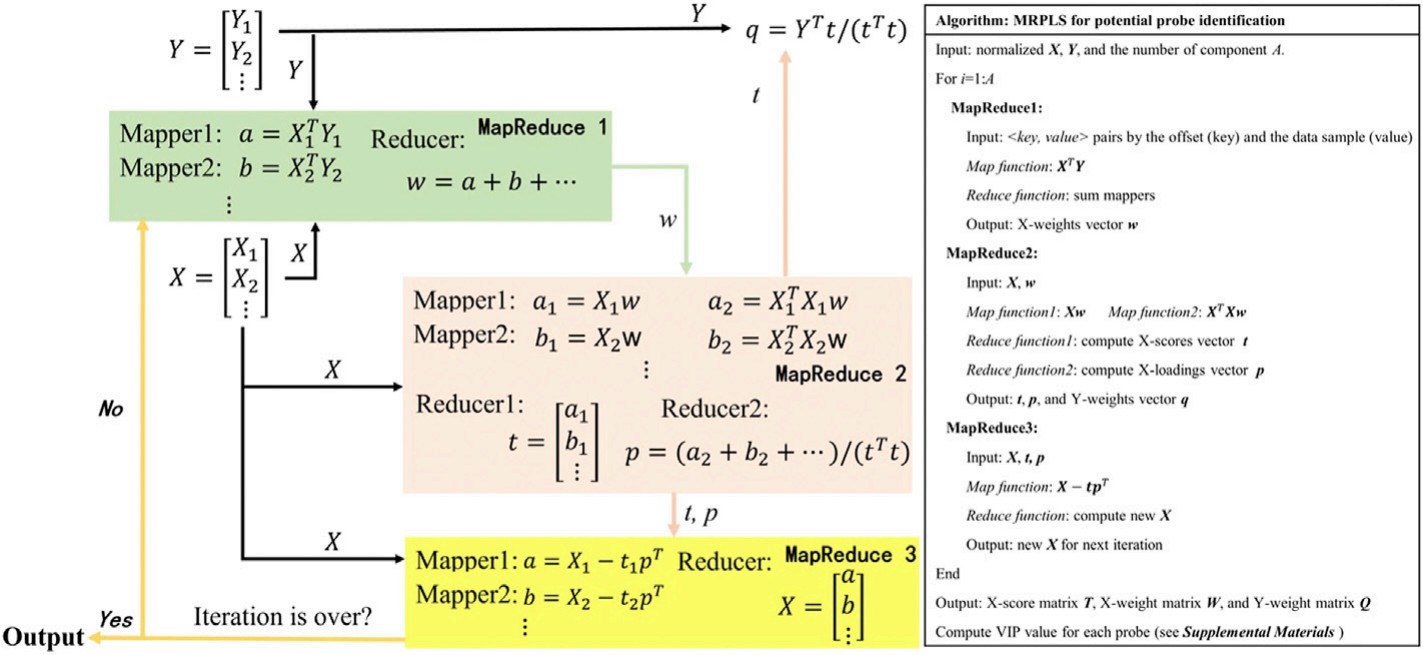
MapReduce-based partial least squares (MRPLS) algorithm flow chart and pseudo-code.

The details of each Map and Reduce function and the corresponding algorithm pipeline and pseudo-codes are shown in Figure 3. The package can be made available on request.

### A modified prognostic index for survival analysis

To integrate both expression and methylation values of genes for each type of cancer, we modified the prognostic index (PI) (Yang et al., 2017) (the original method is described in the Supplementary Material) to include both expression values and methylation levels to evaluate the survival risk of a patient using a multivariate Cox proportional hazard model (R package “survival”):

$${PI}_{i}=\overset{N}{\underset{n=1}{\sum }}\alpha_{n}m_{ni}+\beta_{n}e_{ni},$$ (9)

where *N* is the number of signature genes, $\alpha_{n}$ and $\beta_{n}$ are the regression coefficients of the Cox proportional hazard model for gene *n*, and $m_{ni}$ and $e_{ni}$ are the methylation and gene expression level of gene *n* in sample *i*, respectively. Samples were divided into high- and low-risk groups according to the median PI of the patients in the whole cohort. Then, the Kaplan–Meier (KM) method and log-rank test were used to test the difference between survival risks of these two groups.

### Differential co-expression network inferring method

**FIGURE 4:**
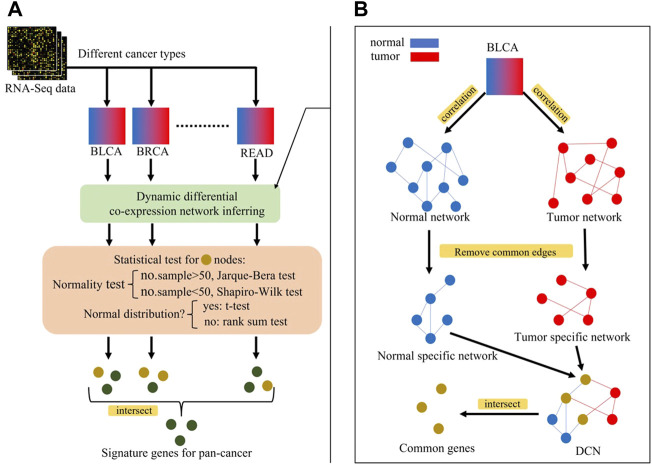
**(A)** Overview of identification process of signature genes for pan-cancer, **(B)** Inferring of differential co-expression network (DCN). Blue nodes represent genes in normal network, red nodes represent genes in tumor network, and yellow nodes represent genes in both networks (intersection). Perform statistical tests on yellow nodes and green nodes represent statistically significant genes, i.e., signature genes for pan-cancer early-stage diagnosis.

The steps for inferring a differential co-expression network (DCN) are shown as follows and in Figure 4B:

⁃ Inferring tumor co-expression network: Pearson’s correlation coefficient (PCC) between any two gene pairs in tumor samples was calculated, and each *p-*value of PCC was corrected by the false discovery rate (FDR). Then, the gene pairs with the corrected *p-*value < 0.05 were chosen as the edges of the tumor co-expression network.
⁃ Inferring normal co-expression network: PCC between any two gene pairs in non-malignant samples was calculated, and each *p-*value of PCC was corrected by the FDR. Then, the gene pairs with the corrected *p-*value < 0.05 were chosen as the edges of the normal co-expression network.
⁃ Selecting the common part between tumor and normal co-expression networks.
⁃ Selecting tumor-specific GRN and normal-specific GRN by removing the common part from the tumor or normal co-expression network, respectively.
⁃ Common genes between the normal-specific and the tumor-specific networks were selected for further statistical tests.
⁃ Checking whether the gene expression in tumor or non-malignant samples was normally distributed: if the number of samples >50, the Jarque–Bera test was used, otherwise the Shapiro–Wilk test was used.
⁃ For normal distribution genes, a two-sided t-test with the corrected *p-*value < 0.05 was used to select genes whose expression values are significantly different between tumor and non-malignant samples. Otherwise, the Wilcoxon rank sum test was used.

After performing the aforementioned process, each type of cancer received a set of genes, which were 1) functionally related both in tumor and non-malignant samples and 2) significantly differentially expressed between tumor and non-malignant samples.

### Identification of signature loci for pan-cancer based on genome-wide methylation and expression data

To overcome the drawbacks caused by the huge difference between the sample number and locus number, to reduce high noise inherent in the genome data, to reduce the false-positive rate, to improve the efficiency in analyzing the big pan-cancer data, and to identify the functionally related real signature methylation loci, a multi-step method of data preprocessing, the MRPLS- and DCN-based procedure was performed and summed up as follows (Figure 1):

1) Data preprocessing using the aforementioned multi-step method;
2) Potential methylation locus identification using MRPLS;
3) Functionally related gene identification using the DCN inferring method followed by statistical tests;
4) Early-stage signature methylation locus identification by mapping these genes back to methylation loci.

The major merit of using MRPLS is that it is a linear method. This is the reason it is used to select highly related methylation loci rather than the final signature loci. The major function of MRPLS is to select methylation loci highly related to pan-cancer early-stage diagnosis effectively and as precisely as possible. Therefore, the following steps 3 and 4 are equally important for real-signature loci identification.

After this procedure, the identified methylation loci were highly related to early-stage tumor diagnosis and prognosis and functionally related to each other through their gene expression levels for pan-cancer.

1) SVM (support vector machine, see Supplementary Material) was used to distinguish early-stage tumor from non-malignant samples using only methylation values of potential methylation loci as input.
2) To verify the roles of early-stage signature methylation loci in tumor diagnosis and prognosis, GO and KEGG (Supplementary Material) pathway enrichment analyses and survival analysis of patients were performed.

It is reasonable to consider them as signature methylation loci that can serve as early-stage diagnosis and prognosis biomarkers.

## Results

### Potential methylation loci identified using MRPLS

To further speed up the calculation and overcome neighborhood co-methylation, the preprocessed genome-wide methylation loci (292,610 loci) were divided into 11 blocks according to their chromosome locations. In this way, the number of loci in each block ranged from 20,000 to 60,000. The details of the 11 blocks are shown in Supplementary Table S1. They were fed into MRPLS in parallel. Then, the top 22,000 loci (smaller than one-tenth of 292,610 loci) with the biggest VIP values were selected for further analysis.

To verify whether these methylation loci were highly related to early-stage tumor diagnosis, their methylation values were used to classify early-stage tumor from non-malignant samples using SVM algorithm with a five-fold cross-validation. If the classification results are good enough, it means the methylation values of these loci contain enough information for early-stage tumor diagnosis. The training dataset consists of 12 types of cancers from TCGA, and the independent test set includes the other six methylation profiles from TCGA and GEO databases (Table 1). The definition of the classification performance measurements is available in the Supplementary Material.

Table 2 shows the classification performance of the training set and independent test set. For the training dataset, we can see that, except for PAAD, the accuracies of all other types of cancer are >90%, and the average accuracy is >95%. Particularly, two types of cancer, CHOL and READ, reach 100%. Given that there are extremely unbalanced sample sizes in BRCA, LIHC, and LUAD (the number of non-malignant samples is much smaller than that of tumor samples), precision, recall, and F1 score (the harmonic average of precision and recall) were used to further evaluate the performance of the classification model. We can see that the averages of these three measurements are all >90%. AUCs are also shown in Table 2 and Supplementary Figure S1A. The average AUC is 0.958, and 5 out of 12 types of cancer even reach 1. Among the 12 types of cancer, only ESCA and PAAD have comparatively poorer results, whose AUCs are <0.90.

**TABLE 2:** Performance of the methylation loci-based diagnostic model for the training set and independent test set.

Cancer	Accuracy	Precision	Recall	F1 score	AUC
Training data set					
BLCA	97.260	100.000	90.476	95.000	0.997
BRCA	99.257	98.947	97.917	98.429	0.999
CHOL	100.000	100.000	100.000	100.000	1.000
COAD	98.765	100.000	97.368	98.667	1.000
ESCA	85.455	90.000	56.250	69.231	0.841
HNSC	93.023	95.833	92.000	93.878	0.973
LIHC	98.722	94.231	98.000	96.078	0.998
LUAD	99.643	96.970	100.000	98.462	1.000
LUSC	99.460	100.000	97.619	98.795	1.000
PAAD	83.871	77.778	70.000	73.684	0.738
PRAD	93.684	88.000	88.000	88.000	0.945
READ	100.000	100.000	100.000	100.000	1.000
Average	95.762	95.147	90.636	92.519	0.958
Independent test set					
GEO-AML	98.649	100.000	96.667	98.305	1.000
GEO-breast	97.500	97.436	95.000	96.203	0.998
GEO-liver	97.727	97.015	98.485	97.744	0.987
TCGA-KIRC	99.683	100.000	99.375	99.687	1.000
TCGA-KIRP	99.528	100.000	97.778	98.876	1.000
TCGA-THCA	97.947	92.983	94.643	93.805	0.979
Average	98.506	97.906	96.991	97.437	0.994

Similarly, for the independent test set, we also obtained good results (Table 2 and Supplementary Figure S1B). For both TCGA and GEO datasets, all measures are >90, and their average values are all >95. In summary, we can conclude that these potential methylation loci selected by MRPLS can successfully diagnose early-stage tumor samples from non-malignant samples for different cancers. Consequently, these loci were proven to be highly related to early-stage pan-cancer.

The 22,000 loci are located at different positions of 4,973 genes. Specifically, 29.37% of them are located at the transcription start site (TSS) and 47.19% of loci are located at the gene body (Supplementary Figure S2A). In addition, 36.5% of loci are distributed on the island, 7.6% on the shelf, and 20.14% on shore (Supplementary Figure S2B).

### Functionally related loci identified for pan-cancer early-stage diagnosis

**FIGURE 5:**
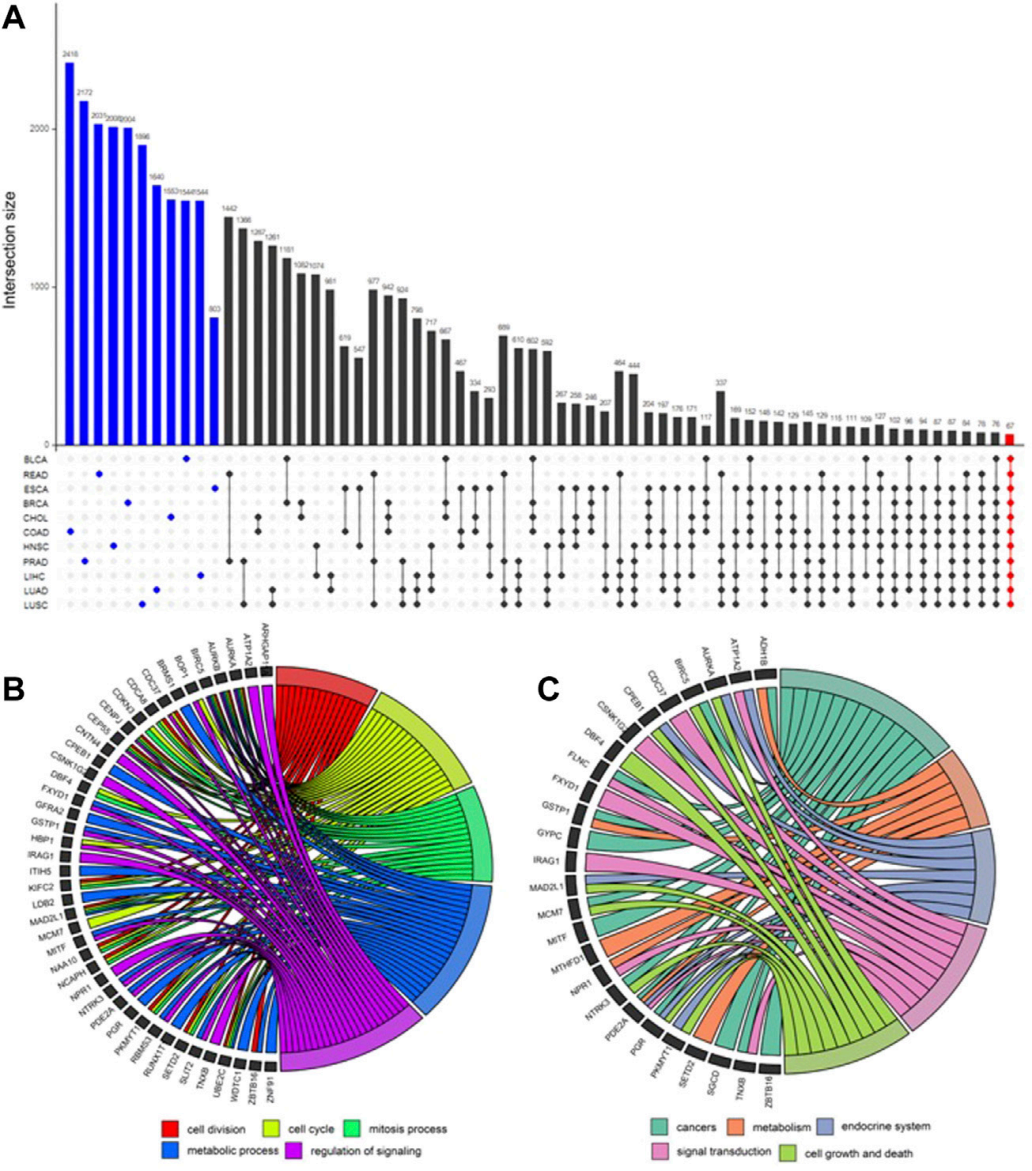
UpSet plot for differential genes’ intersection among 11 cancers and functional enrichment analysis. **(A)** In the UpSet plot, the intersection of different cancers is represented by solid dots and lines, and the number of intersections is represented by the histogram above. Different numbers of significantly differential genes for each cancer (marked blue) were identified. Finally, 67 early-stage pan-cancer biomarkers were identified by taking the intersection of 11 cancers (marked red). **(B)** Gene Ontology (GO) enrichment analysis. The top five significantly enriched GO biological processes and relevant genes. **(C)** Kyoto Encyclopedia of Genes and Genomes (KEGG) pathway enrichment analysis. The top five significantly enriched KEGG pathways and relevant genes.

After obtaining the potential loci, functionally related and differently expressed genes were further identified using DCN inferring method and statistical tests (Figure 4). As shown in Figure 5A, after applying statistical tests, we obtained different numbers of significant genes for different types of cancer. Among them, no such kind of genes could be identified only for PAAD, which may be because of the small sample size of this type of cancer. Then, 67 common genes among the remaining 11 types of cancers were selected as the final signature genes whose methylation variations may be responsible for the early-stage tumor diagnosis and prognosis. The detailed information of these 67 genes and their corresponding methylation loci is summarized in Supplementary Table S2.

GO and KEGG enrichment analyses were applied to these 67 genes, and the results are shown in Figures 5B,C. The top five GO biological processes include “cell division,” “cell cycle,” “mitosis process,” “metabolic process,” and “regulation of signaling.” The top enriched KEGG pathways are “cancers,” “metabolism,” “endocrine system,” “signal transduction,” and “cell growth and death.”

**FIGURE 6:**
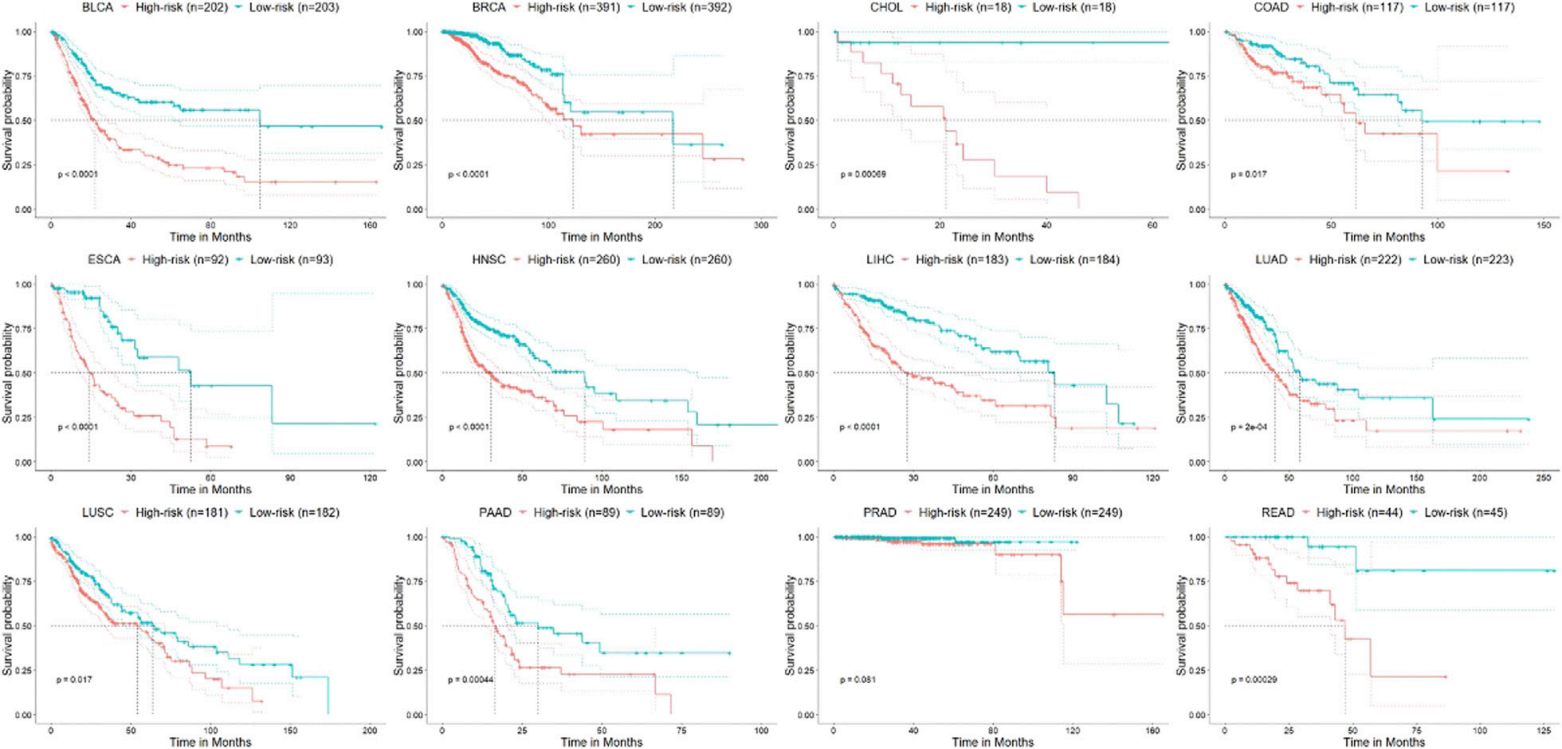
Kaplan–Meier survival curves for overall survival of different types of cancer. Statistical difference in the outcome between high and low PI groups is indicated by the log-rank test *p-*value. “+” stands for the censoring samples.

To further test whether methylation and gene expression levels of the 67 signature genes are clinically related to early-stage tumor patients, survival analysis using a multivariate Cox proportional hazard model was applied to them. KM survival curves are shown in Figure 6. It shows that they are highly related to the survival risk of patients with 10 out of 11 types of cancer (except for PRAD). It strongly proved that they could serve as potential targets for early-stage tumor prognosis.

## Discussion

In the past decade, results have been remarkably accelerated in the validation of the concept that cancer is a disease of epigenetic, as well as genetic, abnormalities. There is even an emerging view on what is now called “the cancer epigenome.” DNA methylation is a major epigenetic modification that is involved in differentiation and development, aging, tumorigenesis, and other diseases. Aberrant methylation, including hypomethylation of oncogenes and hypermethylation of tumor suppressor genes (Irizarry et al., 2009), is a central feature of carcinogenesis and is an early event in many tumors (Luo et al., 2020). All these facts have laid down a solid foundation for identifying methylation loci for pan-cancer early-stage diagnosis.

As mentioned previously, however, the most widely used methylation platform is Infinium HumanMethylation450k. It includes 485,000 CpG loci, which means there are 485,000 variables in methylation data. For the HM850 k platform and other platforms based on the whole-genome bisulfite sequencing (WGBS) technique (Beck et al., 2022), there are even much more loci/variables. One of the most challenging problems is how to efficiently analyze big pan-cancer methylation data. Correspondingly, MapReduce has been taken into consideration to provide fast and cost-effective solutions (Li et al., 2020). The MapReduce framework is a solution originally provided by Google for processing big data in a distributed and parallel way. It is a software framework designed to parallelly run over a cluster of machines/nodes. Up to now, Hadoop, Spark, and even MATLAB provide an integrated MapReduce platform for developers so that they only need to focus on the explicit expressions of “Map” and “Reduce” (Li et al., 2020). It is an excellent framework for processing and analyzing big data. On the other hand, although new non-linear methods [for example, convolutional neural networks (Wang et al., 2021)] have been rapidly developed, PLS is still the most widely used multivariate statistical method considering the model interpretation of it in signature identification. Therefore, the MapReduce-based PLS (MRPLS) was proposed as the first application of a multivariate statistical method in modeling and signature identification with big data. The whole genome-wide methylation loci were divided into 11 blocks and were analyzed with MRPLS parallelly.

Another challenging problem is that it has been reported that methylation of CpGs located on the same DNA fragment occurs non-stochastic. In other words, closer neighboring CpG sites are more likely to share the same methylation status (Affinito et al., 2020). Dividing genome-wide loci into different blocks and to select the most important loci in each block can not only analyze such a big genome-wide methylation data efficiently but also avoid selecting false-positive important loci caused by co-methylation.

MRPLS is supposed to select methylation loci highly related to pan-cancer early-stage diagnosis, and then the DCN and the following steps were supposed to identify functionally related methylation loci from them. Therefore, MRPLS should not be too time-consuming or computation-consuming. PLS has shown its superiority in supervised classification problems in bioinformatics in several studies. Therefore, it was chosen in our study. The basic PLS can be easily extended to other forms such as nonlinear PLS and dynamic PLS.

As a result, 22,000 potential loci were selected. Final real-signature loci would be identified out of these followed by the DCN inferring method. A closer relationship between them for classification would make sure the performance of the final signature loci and reduce the false-positive ratio. The SVM model for classifying early-stage tumor from non-malignant samples using the methylation values of only these 22,000 potential loci strongly proved that their methylation profile contained enough information for early-stage tumor diagnosis.

The third big challenging problem is that it is now a well-recognized fact that the main difference between cancer and normal cells is the complex landscape of genetic and epigenetic aberrations, which usually cause a dynamic rewiring of GRNs at a system level (Hanahan and Weinberg, 2011). There might be inconsistent opinions about the effects of the methylation status on the promoter or other parts of genes, but almost no argument on the opinion that a gene can be directly regulated only by its own methylation status (Matys et al., 2003). A reasonable hypothesis is that the methylation status of signature loci regulates expression values of their own genes, and then these genes are regulated through their expression and functionally work together to initiate tumors. Therefore, 22,000 highly related methylation loci were mapped to 4,973 genes. Then, functionally related genes were identified using the DCN inferring method among these 4,973 genes.

To double-ensure that signature genes are not only functionally related but also significantly differentially expressed between tumor and non-malignant samples, significance tests were performed following the DCN inferring step. It is to be noted that statistical results have a lot to do with the right choice of statistical methods according to the data distribution and sample size (Bandyopadhyay et al., 2014). Specifically, parametric statistical tests require data to be normally distributed while non-parametric tests do not. For normally distributed data, parametric tests usually get better results than non-parametric tests. On the contrary, when data distribution is non-normal, the *p-*value of parametric tests may be misleading and non-parametric methods should be used. Additionally, when the number of samples is less than 50, the aforementioned situation needs to be carefully handled (Mallik et al., 2017). So in our study, for both tumor and non-malignant samples, if the number of samples >50, the Jarque–Bera test was used to check the distribution of the sample, and if the number of samples <50, the Shapiro–Wilk test was used. When both tumor samples and non-malignant samples meet the normal distribution, the two-sided t-test with the corrected *p-*value < 0.05 was used to filter genes; otherwise, the Wilcoxon rank sum test was used.

After performing the aforementioned process, each type of cancer received a statistically significant set of genes. Common genes among all cancers were then selected as the final genes, and their methylation loci were correspondingly selected as the final signature methylation loci for pan-cancer early-stage tumor diagnosis. The details are listed in Supplementary Table S2.

**FIGURE 7:**
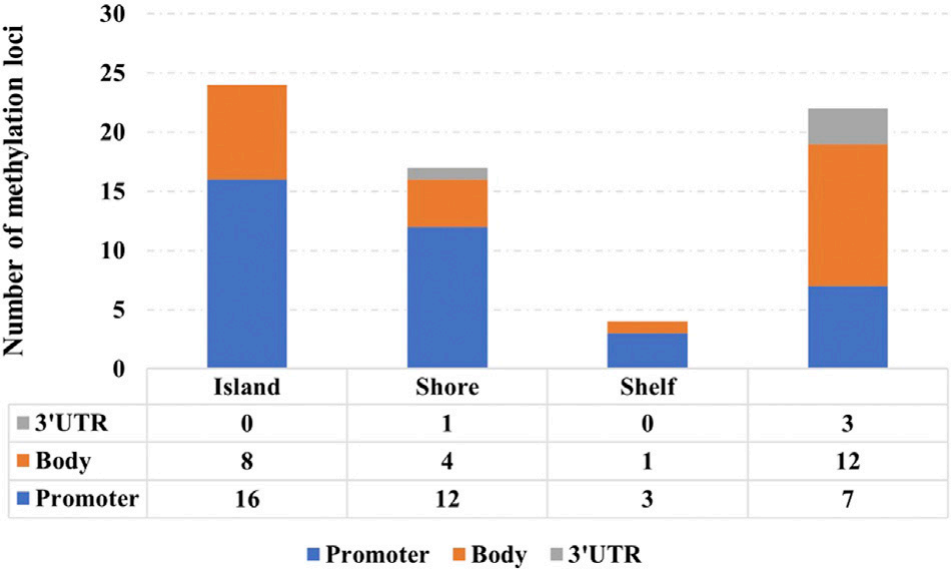
Distribution of 67 signature methylation loci.

Figure 7 shows the distribution of 67 signature methylation loci. Among them, 38 loci are distributed on the promoter region (including TSS1500, TSS200, first exon, and 5′ UTR), 25 loci are distributed on the gene body region, and 4 loci are located at 3′ UTR. One possible explanation for the effect of promoter methylation is that the methylation status affects the binding affinity between transcription factors with cognate DNA sequences (i.e., methylation-sensitive or -resistant), and the regulatory function of the affected transcription factors might be either positive or negative on their target genes (Ma et al., 2013).

Figure 6 shows the KM survival curves with the log-rank test for all 12 training cancer sets. Because of the small size and inherent characteristics of data, PAAD did not obtain any significantly differential genes using the DCN inferring method (see *Methods*). However, from Figure 6, we can see that PAAD patients can still be significantly (*p* = 0.44E-4) divided into high- and low-risk groups by 67 genes that were intersected by other 11 cancers. The result once again proves that these genes we selected can be considered pan-cancer early diagnosis signatures.

On the other hand, among 12 cancers, only PRAD did not get significant results in survival analysis (*p* > 0.05). For PRAD, there are 498 early-stage cancer samples available, of which only 10 patients’ overall survival (OS) status is “deceased” and that of the others is “living.” In other words, the proportion of failure events in PRAD is too low (only 0.02) to get any meaningful results. This phenomenon is in line with the basic laws of survival analysis and has been supported by other published literatures (Zupan et al., 2000; Nezhad et al., 2019).

The biological process of GO refers to biological goals that genes or gene products help to achieve, which is the most important independent ontology that we care most about (Ashburner et al., 2000). The top five remarkably involved biological processes among 67 genes and relevant genes are shown in Figure 5B. These biological processes include “cell division,” “cell cycle,” “mitosis process,” “metabolic process,” and “regulation of signaling,” which have been proved to be highly related to the occurrence and development of a variety of cancers (Susan et al., 1990; Jerby et al., 2012; Williams and Stoeber, 2012; Dominguez-Brauer et al., 2015; Schmid, 2017; Zhang et al., 2020).

A total of 43 genes are enriched in at least one biological process. Among these genes, *AURKA*, *AURKB*, *BIRC5*, *CENPJ*, *CEP55*, *MAD2L1*, and *UBE2C* are enriched in at least four biological processes.

⁃ The latest review by Du et al. (2021) reports that the activation of *AURKA* has been demonstrated to play an important role in a wide range of cancers.
⁃ The research of Bertran-Alamillo et al. (2019) reveals that *AURKB* constitutes a potential target in non-small cell lung cancer (NSCLC) progressing to anti-EGFR therapy and not carrying resistance mutations.
⁃ Gai et al. (2020) used integrative bioinformatics analysis to reveal that *BIRC5* may be adopted as a promising predictive marker and potential therapeutic target in breast cancer.
⁃ Dastsooz et al. (2019) performed a comprehensive bioinformatics analysis and then concluded that *UBE2C* is overexpressed in all 27 cancers they investigated and its expression is significantly higher in late-stage tumors, which might indicate its involvement in tumor progression and invasion.

Figure 5C shows the main enriched KEGG pathways and relevant 26 genes. They are “cancers,” “metabolism,” “endocrine system,” “signal transduction,” and “cell growth and death.” These pathways have also been proved to be highly related to cancers (Gleeson and Shalet, 2004; DeBerardinis et al., 2008; Shimizu et al., 2014; Sever and Brugge, 2015). The other genes of 67 signatures, which have not been verified yet, are likely to be cancer vulnerability genes which are worth further exploring in future studies.

In addition to finding the common signatures for pan-cancer, we further explored the cancer-specific prognostic markers of 67 genes based on the HR of the Cox model. In the Cox model, an HR above 1 indicates a covariate that is positively associated with the event probability and thus negatively associated with the length of survival. In other words, a covariate with HR > 1 is called a bad prognostic factor. Cancer-specific prognostic markers (HR > 1) are summarized for 12 cancers with *p* < 0.05 (Wald test) in Supplementary Table S3. It shows that the number of expression prognostic markers is much more than that of methylation prognostic markers in most cancers, which indicates that gene expression plays more direct roles in cancer initiation than methylation does. It also proved that methylation can only function by regulating its own gene expression status.

Moreover, the main focus of this study was to propose an interpretable method for analyzing big data cost-effectively. Thus, the terms of nonlinearity or non-Gaussian distribution have not been considered in this study. Because methylation data measured by HM450k have been the most widely used data currently, they were used as an example in our study. However, based on the proposed modeling framework, other big data (i.e., HM850k) can also be analyzed using our MRPLS method. The basic PLS can be easily extended to other forms such as nonlinear PLS and dynamic PLS.

## Conclusion

In this study, we developed a new MapReduce-based PLS method for analyzing methylation data parallelly and efficiently to overcome the “curse of big data” brought up by pan-cancer studies and the false-positive caused by neighboring co-methylation. We analyzed methylation and expression profiles of 12 cancers from TCGA and identified 67 signature methylation loci and corresponding genes for early-stage pan-cancer diagnosis and prognosis. Their methylation status and the difference in their co-expression network and expression values were all highly related to early-stage tumors and non-malignant sample classification. The biological processes and pathways they were significantly involved in were proved to play key roles in cancer initiation. Most importantly, their methylation and expression values are highly related to patient survival risk. Furthermore, the selected biomarkers could provide a reliable reference for understanding cancer progress mechanisms and precision medicine. The data analysis workflow that we proposed could be applied to any large-scale biological data for integrative signature discovery.

## Data Availability

Publicly available datasets were analyzed in this study. These data can be found at: level-3 DNA methylation (HumanMethylation450), level-3 RNA-Seq V2, and clinical data were downloaded from TCGA. The data of the other three types of cancer from TCGA and three GEO datasets (GSE54503, GSE63409, and GSE66695) were collected as the independent validation dataset.
