# Supplementary material for: DNA methylation loci identification for pan-cancer early-stage diagnosis and prognosis using a new distributed parallel partial least squares method
Source: Front Genet. 2022 Oct 19;13:940214. doi: 10.3389/fgene.2022.940214 (PMC9626520; doi:10.3389/fgene.2022.940214)
Supplement: Supplementary file 1 [file DataSheet1.PDF]

Supplemental Materials for:

Distributed parallel PLS for signature DNA methylation loci identification of early-stage diagnosis and prognosis with big pan-cancer data

Qi-en He[a], Jun-xuan Zhu[a], Li-yan Wang[a], En-ci Ding[b], Kai Song[a]*

[a]School of Chemical Engineering & Technology, Tianjin University, Tianjin, 300072, PR China

[b]Tianjin First Central Hostipal, Tianjin, 300190, PR China

*Corresponding author: Kai Song

Address: School of Chemical Engineering & Technology, Tianjin University, Tianjin, 300072, PR China

Tel: +86 18920317821

E-mail: ksong@tju.edu.cn

Partial least squares

PLS is a widely used algorithm for modeling relationships between sets of observed variables using latent variables. It comprises regression and classification tasks as well as dimension reducing and modeling. Although PLS is not originally designed as a tool for statistical discrimination, applied scientists routinely use PLS for classification and there is substantial empirical evidence to suggest that it performs well in that role.[1] The origins of PLS are traced back to Wold's original non-linear iterative partial least squares (NIPALS) algorithm,[2] which is the core of PLS for both classification and regression tasks. Given normalized $\boldsymbol{X}$, $\boldsymbol{Y}$, and the number of component A, for the first component:

(a) Get a starting vector of $\boldsymbol{u}$, here $\boldsymbol{u}$=$\boldsymbol{Y}$.
(b) Calculate X-weights, $\boldsymbol{w}$: $\boldsymbol{w} = \boldsymbol{X}'\boldsymbol{u}/\boldsymbol{u}'\boldsymbol{u}$, then norm $\boldsymbol{w}$ to $||\boldsymbol{w}||$=1.
(c) Calculate X-scores, $\boldsymbol{t}$: $\boldsymbol{t}$=$\boldsymbol{Xw}$.
(d) Calculate X-loadings, $\boldsymbol{p}$: $\boldsymbol{p} = \boldsymbol{X}'\boldsymbol{t}/\boldsymbol{t}'\boldsymbol{t}$.
(e) Calculate Y-weights, $\boldsymbol{q}$: $\boldsymbol{q} = \boldsymbol{Y}'\boldsymbol{t}/\boldsymbol{t}'\boldsymbol{t}$.
(f) Remove the present component from $\boldsymbol{X}$, i.e., $\boldsymbol{X} = \boldsymbol{X} - \boldsymbol{tp}'$, and use this deflated matrix as $\boldsymbol{X}$ in the next component.
(g) Continue with next component (back to (a)) until there is no more significant information in $\boldsymbol{X}$ about $\boldsymbol{Y}$.

Survival analysis

The prognostic index (PI) used in this study to generate high- and low-risk patient groups is modified according to the PI of Yang et al.[3] They only used DNA methylation data to generate PI value for each sample:

$$PI_i = \sum_{n=1}^{N} \alpha_n m_{ni} \tag{S4}$$

where N is the number of signature genes, α_n is the regression coefficients of Cox proportional hazard model for gene n, m_{ni} is the methylation level of gene n in sample i.

Support vector machine (SVM)

SVM is a supervised machine learning method which has been widely used for classification and regression analysis. The key concept of it is non-linear mapping input data into a very high-dimension feature space $\boldsymbol{Z}$, where a linear decision surface is constructed with special properties to ensure the high generalization ability of the model.[4] It is known for having good performance on small sample dataset. The basic idea of SVM can be descripted as:

$$\min_{w,b} \frac{1}{2} ||\boldsymbol{w}||^2 \qquad (2)$$
$$\text{s.t.}\, y_i(\boldsymbol{w}^T \boldsymbol{x}_i + b) \geq 1$$

where $\boldsymbol{x}$ is input data, y_i is ith sample's label, $\boldsymbol{w}$ is the normal vector of hyperplane, and b is displacement term. In this study, SVM classification was implemented using a "LIBSVM" MATLAB package.

Gene ontology (GO) and Kyoto Encyclopedia of Genes and Genomes (KEGG) pathway enrichment analysis

To annotate functional progress involving the signature genes, GO biological process[5] and KEGG pathway enrichment analyzes[6] were carried out using R package "clusterProfiler"[7] and visualized by chord diagram using R package "Goplot".

Classification performance metrics.

Confusion matrix:

**Confusion matrix**		Predicted Class	
		Predicted Value: **Positive** (+)	Predicted Value: **Negative** (-)
True Class	True Value: **Positive** (+)	**TP** True Positive	**FN** False Negative
	True Value: **Negative** (-)	**FP** False Positive	**TN** True Negative

- Accuracy:

$$ACC = \frac{TP + TN}{TP + TN + FP + FN}$$

- Precision:

$$P = \frac{TP}{TP + FP}$$

- Recall (Sensitivity):

$$R = \frac{TP}{TP + FN}$$

- F1score:

$$F1 = \frac{2 * precision * recall}{precision + recall}$$

- Receiver operating characteristic (ROC): Abscissa is false positive rate (FPR), and ordinate is true positive rate (TPR).

$$FPR = \frac{FP}{FP + TN}$$

$$TPR = \frac{TP}{TP + FN}$$

- The area under receiver operating characteristic (ROC) curve (AUC).

Table S1. The distribution of loci on chromosomes and 11 blocks.

Chromosomes	The number of loci	Blocks
1	27715	block1: ch1
2	21105	block2: ch2&ch9
3	14586	block3: ch3&ch8
4	12043	block4: ch4&ch10
5	15042	block5: ch5&ch18&ch20
6	21783	block6: ch6&ch22
7	18600	block7: ch7&ch14
8	12796	block8: ch11&ch15
9	6143	block9: ch12&ch13&Y&multi
10	15339	block10: ch16&ch19
11	17692	block11: h17&ch20&chX
12	14284	
13	7598	
14	8993	
15	9231	
16	13290	
17	16815	
18	3563	
19	14391	
20	6188	
21	2668	
22	4849	
X	7826	
Y	36	
Multi	34	

Table S2-1. The detail information of 67 signature methylation loci and genes for pan-cancer (General information).

Gene symbol	Ensembl	Loci	Chromosome	UCSC_RefGene_Group	Relation_to_UCSC_CpG_Island
ACAN	ENSG00000157766	cg02401352	15	5'UTR	
ADH1B	ENSG00000196616	cg01151642	4	Body	
ARHGAP11A	ENSG00000198826	cg00297451	15	5'UTR	S_Shore
ATP1A2	ENSG00000018625	cg01448881	1	TSS1500	
AURKA	ENSG00000087586	cg01596892	20	TSS200	Island
AURKB	ENSG00000178999	cg02245743	17	TSS200	S_Shore
BIRC5	ENSG00000089685	cg00017271	17	3'UTR	
BOP1	ENSG00000261236	cg02873427	8	Body	Island
BRMS1	ENSG00000174744	cg02025157	11	TSS200	Island
CDC37	ENSG00000105401	cg05357108	19	Body	N_Shore
CDCA8	ENSG00000134690	cg03443676	1	3'UTR	
CDKN3	ENSG00000100526	cg00649500	14	TSS1500	N_Shore
CENPJ	ENSG00000151849	cg04629247	13	5'UTR	N_Shelf
CEP55	ENSG00000138180	cg04026927	10	Body	
CNTN4	ENSG00000144619	cg00912625	3	TSS1500	Island
CPEB1	ENSG00000214575	cg00317577	15	Body	
CSNK1G2	ENSG00000133275	cg01335597	19	5'UTR	N_Shelf
DBF4	ENSG00000006634	cg00663797	7	TSS1500	Island
FAM83H	ENSG00000180921	cg00238815	8	3'UTR	N_Shore
FBXL19	ENSG00000099364	cg00975115	16	Body	Island
FILIP1	ENSG00000118407	cg02586023	6	5'UTR	
FLNC	ENSG00000128591	cg00734629	7	Body	Island
FXYD1	ENSG00000266964	cg03078169	19	5'UTR	N_Shelf
GFRA2	ENSG00000168546	cg00137611	8	Body	N_Shore
GIMAP1	ENSG00000213203	cg01640506	7	Body	Island
GSTP1	ENSG00000084207	cg02659086	11	TSS200	Island

GYPC	ENSG00000136732	cg00014112	2	TSS200	N_Shore
HBP1	ENSG00000105856	cg01353569	7	5'UTR	Island
HSPB6	ENSG00000004776	cg00286125	19	Body	Island
ISG20L2	ENSG00000143319	cg00341602	1	Body	N_Shelf
ITIH5	ENSG00000123243	cg00244882	10	Body	
KCNT2	ENSG00000162687	cg01768775	1	TSS1500	S_Shore
KIFC2	ENSG00000167702	cg01590314	8	TSS200	N_Shore
LDB2	ENSG00000169744	cg02708121	4	Body	
MAD2L1	ENSG00000164109	cg00680768	4	TSS200	Island
MALL	ENSG00000144063	cg00238570	2	Body	N_Shore
MCM7	ENSG00000166508	cg00094751	7	Body	
MEX3A	ENSG00000254726	cg00660163	1	TSS1500	Island
MITF	ENSG00000187098	cg00130817	3	Body	
MRVI1	ENSG00000072952	cg00450571	11	TSS1500	
MTHFD1	ENSG00000100714	cg00691969	14	Body	S_Shore
NAA10	ENSG00000102030	cg00072288	X	TSS200	Island
NALCN	ENSG00000102452	cg01105058	13	TSS1500	Island
NCAPH	ENSG00000121152	cg01906055	2	TSS1500	N_Shore
NPR1	ENSG00000169418	cg00500892	1	TSS1500	Island
NTRK3	ENSG00000140538	cg00145961	15	Body	
PDE2A	ENSG00000186642	cg00239835	11	5'UTR	
PGR	ENSG00000082175	cg00173799	11	TSS1500	S_Shore
PKMYT1	ENSG00000127564	cg00319761	16	1stExon	Island
PPIL1	ENSG00000137168	cg01910741	6	TSS1500	S_Shore
PTGDR	ENSG00000168229	cg02191312	14	TSS200	Island
RBMS3	ENSG00000144642	cg00807684	3	Body	
RUNX1T1	ENSG00000079102	cg00045118	8	Body	
SCARA5	ENSG00000168079	cg00138766	8	3'UTR	

SETD2	ENSG00000181555	cg01702820	3	TSS200	Island
SGCD	ENSG00000170624	cg01474424	5	1stExon	
SLBP	ENSG00000163950	cg00003091	4	TSS1500	S_Shore
SLIT2	ENSG00000145147	cg03260566	4	Body	Island
SNRPD1	ENSG00000167088	cg12351856	18	1stExon	Island
SYNE1	ENSG00000131018	cg00041666	6	Body	
TDRD10	ENSG00000163239	cg00006198	1	5'UTR	S_Shore
TNXB	ENSG00000168477	cg00013543	6	Body	
UBE2C	ENSG00000175063	cg00242976	20	TSS1500	N_Shore
WDTC1	ENSG00000142784	cg00757789	1	TSS1500	Island
YDJC	ENSG00000161179	cg01307115	22	Body	Island
ZBTB16	ENSG00000109906	cg01294198	11	Body	Island
ZNF91	ENSG00000167232	cg02747612	19	TSS1500	

Table S2-2. The detail information of 67 signature methylation loci and genes for pan-cancer (Percentage of Hyper vs Hypo).

Gene symbol	READ		LUAD		PRAD		LUSC		LIHC		HNSC		ESCA		COAD		CHOL		BRCA		BLCA	
	Hyper	Hypo	Hyper	Hypo	Hyper	Hypo	Hyper	Hypo	Hyper	Hypo	Hyper	Hypo	Hyper	Hypo	Hyper	Hypo	Hyper	Hypo	Hyper	Hypo	Hyper	Hypo
ACAN	0.00	100.00	0.00	100.00	0.00	100.00	0.00	100.00	0.00	100.00	0.00	100.00	0.00	100.00	0.00	100.00	0.00	100.00	0.00	100.00	0.00	100.00
ADH1B	0.00	100.00	27.02	72.98	63.57	36.43	10.49	89.51	38.02	61.98	5.56	94.44	7.69	92.31	4.65	95.35	57.14	42.86	51.30	48.70	28.85	71.15
ARHGAP11A	0.00	100.00	0.00	100.00	0.00	100.00	0.00	100.00	0.00	100.00	0.00	100.00	0.00	100.00	0.00	100.00	0.00	100.00	0.00	100.00	0.00	100.00
ATP1A2	60.00	40.00	30.24	69.76	85.71	14.29	25.17	74.83	39.54	60.46	11.11	88.89	23.08	76.92	44.19	55.81	67.86	32.14	83.12	16.88	53.85	46.15
AURKA	0.00	100.00	0.00	100.00	0.00	100.00	0.00	100.00	0.00	100.00	0.00	100.00	0.00	100.00	0.00	100.00	0.00	100.00	0.00	100.00	0.00	100.00
AURKB	0.00	100.00	0.00	100.00	0.00	100.00	0.00	100.00	0.00	100.00	0.00	100.00	0.00	100.00	0.00	100.00	0.00	100.00	0.00	100.00	0.00	100.00
BIRC5	80.00	20.00	79.44	20.56	42.86	57.14	77.62	22.38	89.35	10.65	86.11	13.89	69.23	30.77	83.72	16.28	96.43	3.57	70.78	29.22	88.46	11.54
BOP1	0.00	100.00	0.00	100.00	0.00	100.00	0.00	100.00	0.00	100.00	0.00	100.00	0.00	100.00	0.00	100.00	0.00	100.00	0.00	100.00	0.00	100.00
BRMS1	0.00	100.00	0.00	100.00	0.00	100.00	0.00	100.00	0.00	100.00	0.00	100.00	0.00	100.00	0.00	100.00	0.00	100.00	0.00	100.00	0.00	100.00
CDC37	70.00	30.00	91.94	8.06	75.71	24.29	93.71	6.29	74.52	25.48	100.00	0.00	82.05	17.95	90.70	9.30	78.57	21.43	79.22	20.78	84.62	15.38
CDCA8	0.00	100.00	0.00	100.00	0.00	100.00	0.00	100.00	0.00	100.00	0.00	100.00	0.00	100.00	0.00	100.00	0.00	100.00	0.00	100.00	0.00	100.00
CDKN3	100.00	0.00	99.60	0.40	100.00	0.00	95.10	4.90	99.62	0.38	83.33	16.67	92.31	7.69	100.00	0.00	96.43	3.57	99.68	0.32	96.15	3.85
CENPJ	0.00	100.00	0.00	100.00	0.00	100.00	0.00	100.00	0.00	100.00	0.00	100.00	0.00	100.00	0.00	100.00	0.00	100.00	0.00	100.00	0.00	100.00
CEP55	80.00	20.00	76.21	23.79	6.43	93.57	93.01	6.99	78.33	21.67	88.89	11.11	89.74	10.26	93.02	6.98	25.00	75.00	69.81	30.19	96.15	3.85
CNTN4	0.00	100.00	0.00	100.00	0.00	100.00	0.00	100.00	0.38	99.62	0.00	100.00	0.00	100.00	2.33	97.67	0.00	100.00	0.00	100.00	0.00	100.00
CPEB1	0.00	100.00	0.81	99.19	33.57	66.43	8.39	91.61	3.80	96.20	5.56	94.44	10.26	89.74	0.00	100.00	53.57	46.43	45.78	54.22	3.85	96.15
CSNK1G2	100.00	0.00	98.79	1.21	100.00	0.00	93.01	6.99	100.00	0.00	91.67	8.33	92.31	7.69	97.67	2.33	100.00	0.00	98.38	1.62	98.08	1.92
DBF4	0.00	100.00	0.00	100.00	0.00	100.00	0.00	100.00	0.00	100.00	0.00	100.00	0.00	100.00	0.00	100.00	0.00	100.00	0.00	100.00	0.00	100.00
FAM83H	90.00	10.00	81.45	18.55	95.00	5.00	95.10	4.90	90.87	9.13	100.00	0.00	92.31	7.69	90.70	9.30	96.43	3.57	93.83	6.17	88.46	11.54
FBXL19	100.00	0.00	99.19	0.81	100.00	0.00	99.30	0.70	100.00	0.00	100.00	0.00	100.00	0.00	100.00	0.00	100.00	0.00	99.68	0.32	100.00	0.00
FILIP1	100.00	0.00	79.84	20.16	100.00	0.00	97.90	2.10	60.84	39.16	94.44	5.56	84.62	15.38	95.35	4.65	100.00	0.00	99.03	0.97	100.00	0.00
FLNC	0.00	100.00	0.00	100.00	6.43	93.57	1.40	98.60	9.13	90.87	0.00	100.00	2.56	97.44	9.30	90.70	14.29	85.71	6.17	93.83	9.62	90.38
FXYD1	0.00	100.00	0.40	99.60	0.00	100.00	0.00	100.00	0.38	99.62	0.00	100.00	0.00	100.00	4.65	95.35	14.29	85.71	1.95	98.05	0.00	100.00
GFRA2	0.00	100.00	0.00	100.00	9.29	90.71	0.00	100.00	1.14	98.86	0.00	100.00	2.56	97.44	2.33	97.67	7.14	92.86	9.42	90.58	0.00	100.00

GIMAP1	100.00	0.00	93.55	6.45	97.86	2.14	96.50	3.50	98.10	1.90	100.00	0.00	94.87	5.13	100.00	0.00	96.43	3.57	83.77	16.23	90.38	9.62
GSTP1	0.00	100.00	0.00	100.00	0.71	99.29	0.00	100.00	1.90	98.10	0.00	100.00	0.00	100.00	0.00	100.00	0.00	100.00	0.00	100.00	0.00	100.00
GYPC	10.00	90.00	0.00	100.00	0.00	100.00	0.00	100.00	0.00	100.00	0.00	100.00	0.00	100.00	2.33	97.67	0.00	100.00	0.32	99.68	1.92	98.08
HBP1	0.00	100.00	0.00	100.00	0.00	100.00	0.00	100.00	0.00	100.00	0.00	100.00	0.00	100.00	0.00	100.00	0.00	100.00	0.00	100.00	0.00	100.00
HSPB6	0.00	100.00	0.00	100.00	0.71	99.29	0.00	100.00	8.75	91.25	0.00	100.00	0.00	100.00	0.00	100.00	17.86	82.14	6.17	93.83	1.92	98.08
ISG20L2	0.00	100.00	0.00	100.00	0.00	100.00	0.00	100.00	0.00	100.00	0.00	100.00	0.00	100.00	0.00	100.00	0.00	100.00	0.00	100.00	0.00	100.00
ITIH5	0.00	100.00	0.00	100.00	0.00	100.00	0.00	100.00	0.76	99.24	0.00	100.00	0.00	100.00	11.63	88.37	0.00	100.00	0.32	99.68	0.00	100.00
KCNT2	0.00	100.00	0.00	100.00	0.00	100.00	0.00	100.00	0.00	100.00	0.00	100.00	0.00	100.00	0.00	100.00	0.00	100.00	0.00	100.00	1.92	98.08
KIFC2	0.00	100.00	0.00	100.00	0.00	100.00	0.00	100.00	0.00	100.00	0.00	100.00	0.00	100.00	0.00	100.00	0.00	100.00	0.00	100.00	0.00	100.00
LDB2	20.00	80.00	12.50	87.50	22.86	77.14	16.78	83.22	19.01	80.99	30.56	69.44	15.38	84.62	18.60	81.40	64.29	35.71	24.35	75.65	9.62	90.38
MAD2L1	0.00	100.00	0.00	100.00	0.00	100.00	0.00	100.00	0.00	100.00	0.00	100.00	0.00	100.00	0.00	100.00	0.00	100.00	0.00	100.00	0.00	100.00
MALL	0.00	100.00	2.42	97.58	80.00	20.00	23.78	76.22	10.65	89.35	8.33	91.67	5.13	94.87	2.33	97.67	57.14	42.86	56.49	43.51	3.85	96.15
MCM7	100.00	0.00	97.58	2.42	100.00	0.00	100.00	0.00	100.00	0.00	100.00	0.00	100.00	0.00	97.67	2.33	100.00	0.00	100.00	0.00	100.00	0.00
MEX3A	0.00	100.00	0.00	100.00	0.00	100.00	0.00	100.00	0.00	100.00	0.00	100.00	0.00	100.00	0.00	100.00	0.00	100.00	0.00	100.00	0.00	100.00
MITF	90.00	10.00	98.39	1.61	99.29	0.71	100.00	0.00	91.63	8.37	97.22	2.78	100.00	0.00	97.67	2.33	89.29	10.71	97.73	2.27	96.15	3.85
MRVI1	100.00	0.00	97.58	2.42	100.00	0.00	98.60	1.40	92.78	7.22	91.67	8.33	97.44	2.56	100.00	0.00	71.43	28.57	98.05	1.95	100.00	0.00
MTHFD1	0.00	100.00	0.00	100.00	0.00	100.00	0.00	100.00	0.00	100.00	0.00	100.00	0.00	100.00	0.00	100.00	0.00	100.00	0.00	100.00	0.00	100.00
NAA10	0.00	100.00	0.00	100.00	0.00	100.00	0.00	100.00	0.00	100.00	0.00	100.00	0.00	100.00	0.00	100.00	0.00	100.00	0.00	100.00	0.00	100.00
NALCN	0.00	100.00	0.00	100.00	0.00	100.00	0.00	100.00	0.38	99.62	0.00	100.00	0.00	100.00	0.00	100.00	0.00	100.00	0.32	99.68	0.00	100.00
NCAPH	0.00	100.00	6.85	93.15	19.29	80.71	14.69	85.31	61.60	38.40	13.89	86.11	7.69	92.31	6.98	93.02	35.71	64.29	13.31	86.69	9.62	90.38
NPR1	0.00	100.00	0.00	100.00	0.00	100.00	0.00	100.00	0.00	100.00	0.00	100.00	0.00	100.00	0.00	100.00	0.00	100.00	0.00	100.00	0.00	100.00
NTRK3	60.00	40.00	58.47	41.53	88.57	11.43	39.16	60.84	37.64	62.36	52.78	47.22	43.59	56.41	74.42	25.58	92.86	7.14	87.99	12.01	30.77	69.23
PDE2A	0.00	100.00	0.00	100.00	0.71	99.29	0.00	100.00	4.18	95.82	0.00	100.00	0.00	100.00	0.00	100.00	10.71	89.29	1.62	98.38	0.00	100.00
PGR	0.00	100.00	0.00	100.00	0.00	100.00	0.00	100.00	0.00	100.00	0.00	100.00	0.00	100.00	0.00	100.00	0.00	100.00	0.00	100.00	0.00	100.00
PKMYT1	0.00	100.00	0.00	100.00	0.00	100.00	0.00	100.00	0.00	100.00	0.00	100.00	0.00	100.00	0.00	100.00	0.00	100.00	0.00	100.00	0.00	100.00
PPIL1	0.00	100.00	4.44	95.56	0.71	99.29	9.09	90.91	28.90	71.10	36.11	63.89	17.95	82.05	4.65	95.35	14.29	85.71	14.29	85.71	5.77	94.23
PTGDR	0.00	100.00	0.00	100.00	0.00	100.00	0.00	100.00	0.00	100.00	0.00	100.00	0.00	100.00	0.00	100.00	0.00	100.00	0.32	99.68	1.92	98.08
RBMS3	0.00	100.00	0.00	100.00	0.00	100.00	0.00	100.00	0.00	100.00	0.00	100.00	0.00	100.00	0.00	100.00	0.00	100.00	0.00	100.00	0.00	100.00
RUNX1T1	10.00	90.00	8.06	91.94	37.14	62.86	2.10	97.90	1.52	98.48	0.00	100.00	5.13	94.87	13.95	86.05	57.14	42.86	14.29	85.71	26.92	73.08

SCARA5	100.00	0.00	94.35	5.65	99.29	0.71	95.80	4.20	70.72	29.28	94.44	5.56	89.74	10.26	93.02	6.98	75.00	25.00	98.70	1.30	92.31	7.69
SETD2	0.00	100.00	0.00	100.00	0.00	100.00	0.00	100.00	0.00	100.00	0.00	100.00	0.00	100.00	0.00	100.00	0.00	100.00	0.00	100.00	0.00	100.00
SGCD	0.00	100.00	0.00	100.00	2.14	97.86	0.00	100.00	2.66	97.34	0.00	100.00	0.00	100.00	0.00	100.00	3.57	96.43	2.60	97.40	5.77	94.23
SLBP	0.00	100.00	0.00	100.00	0.00	100.00	0.00	100.00	0.00	100.00	0.00	100.00	0.00	100.00	0.00	100.00	0.00	100.00	0.00	100.00	0.00	100.00
SLIT2	20.00	80.00	0.81	99.19	20.71	79.29	2.10	97.90	6.84	93.16	0.00	100.00	5.13	94.87	20.93	79.07	25.00	75.00	12.66	87.34	50.00	50.00
SNRPD1	0.00	100.00	0.00	100.00	0.00	100.00	0.00	100.00	0.00	100.00	0.00	100.00	0.00	100.00	0.00	100.00	0.00	100.00	0.00	100.00	0.00	100.00
SYNE1	0.00	100.00	0.00	100.00	0.00	100.00	0.00	100.00	0.00	100.00	0.00	100.00	0.00	100.00	2.33	97.67	0.00	100.00	1.95	98.05	0.00	100.00
TDRD10	10.00	90.00	47.58	52.42	74.29	25.71	49.65	50.35	72.24	27.76	58.33	41.67	38.46	61.54	16.28	83.72	50.00	50.00	77.60	22.40	34.62	65.38
TNXB	0.00	100.00	0.00	100.00	0.00	100.00	0.00	100.00	0.00	100.00	0.00	100.00	0.00	100.00	0.00	100.00	0.00	100.00	0.00	100.00	0.00	100.00
UBE2C	0.00	100.00	0.00	100.00	0.00	100.00	0.00	100.00	2.66	97.34	0.00	100.00	0.00	100.00	0.00	100.00	0.00	100.00	0.00	100.00	0.00	100.00
WDTC1	0.00	100.00	0.00	100.00	0.00	100.00	0.00	100.00	0.00	100.00	0.00	100.00	0.00	100.00	0.00	100.00	0.00	100.00	0.00	100.00	0.00	100.00
YDJC	40.00	60.00	44.76	55.24	85.71	14.29	35.66	64.34	76.81	23.19	61.11	38.89	51.28	48.72	41.86	58.14	92.86	7.14	71.10	28.90	36.54	63.46
ZBTB16	100.00	0.00	95.56	4.44	100.00	0.00	90.21	9.79	99.62	0.38	94.44	5.56	87.18	12.82	97.67	2.33	100.00	0.00	96.43	3.57	96.15	3.85
ZNF91	40.00	60.00	56.45	43.55	55.00	45.00	44.76	55.24	45.63	54.37	52.78	47.22	25.64	74.36	34.88	65.12	0.00	100.00	31.49	68.51	38.46	61.54

Table S2-3. The detail information of 67 signature methylation loci and genes for pan-cancer (Up/down regulated vs non-malignant samples).

Gene Symbol	BLCA	BRCA	CHOL	COAD	ESCA	HNSC	LIHC	LUAD	LUSC	PRAD	READ
ACAN	UP	UP	UP	UP	UP	UP	UP	UP	Normal	UP	UP
ADH1B	DOWN	DOWN	DOWN	DOWN	DOWN	DOWN	DOWN	DOWN	DOWN	DOWN	DOWN
ARHGAP11A	UP	UP	UP	Normal	UP	UP	UP	UP	UP	UP	NS
ATP1A2	DOWN	DOWN	DOWN	DOWN	DOWN	DOWN	UP	DOWN	DOWN	DOWN	DOWN
AURKA	UP	UP	UP	UP	UP	UP	UP	UP	UP	UP	UP
AURKB	UP	UP	UP	UP	UP	UP	UP	UP	UP	UP	UP
BIRC5	UP	UP	UP	UP	UP	UP	UP	UP	UP	UP	UP
BOP1	UP	UP	NS	UP	UP	UP	UP	UP	UP	UP	UP
BRMS1	UP	UP	UP	Normal	Normal	UP	UP	Normal	UP	Normal	NS
CDC37	NS	UP	Normal	NS	Normal	NS	Normal	Normal	Normal	Normal	NS
CDCA8	UP	UP	UP	UP	UP	UP	UP	UP	UP	UP	UP
CDKN3	UP	UP	UP	UP	UP	UP	UP	UP	UP	UP	UP
CENPJ	NS	NS	NS	Normal	UP	Normal	UP	UP	UP	Normal	Normal
CEP55	UP	UP	UP	UP	UP	UP	UP	UP	UP	UP	UP
CNTN4	DOWN	Normal	DOWN	DOWN	DOWN	DOWN	DOWN	DOWN	DOWN	Normal	DOWN
CPEB1	DOWN	DOWN	UP	DOWN	DOWN	NS	NS	DOWN	DOWN	DOWN	DOWN
CSNK1G2	Normal	UP	NS	Normal	Normal	Normal	NS	NS	UP	UP	UP
DBF4	UP	UP	NS	UP	UP	UP	UP	UP	UP	UP	UP
FAM83H	NS	UP	UP	UP	Normal	Normal	UP	UP	UP	UP	UP
FBXL19	UP	UP	UP	NS	Normal	UP	Normal	UP	UP	UP	UP
FILIP1	DOWN	DOWN	DOWN	DOWN	DOWN	DOWN	Normal	DOWN	DOWN	DOWN	DOWN
FLNC	DOWN	DOWN	UP	DOWN	DOWN	DOWN	UP	Normal	DOWN	DOWN	DOWN
FXYD1	DOWN	DOWN	DOWN	DOWN	DOWN	DOWN	DOWN	DOWN	DOWN	DOWN	DOWN
GFRA2	DOWN	DOWN	DOWN	DOWN	DOWN	DOWN	DOWN	DOWN	DOWN	DOWN	DOWN
GIMAP1	DOWN	DOWN	DOWN	DOWN	DOWN	Normal	Normal	DOWN	DOWN	NS	DOWN
GSTP1	Normal	NS	UP	UP	UP	NS	DOWN	Normal	UP	DOWN	UP

GYPC	DOWN	DOWN	UP	DOWN	DOWN	DOWN	DOWN	DOWN	DOWN	DOWN	DOWN
HBP1	DOWN	Normal	Normal	DOWN	DOWN	DOWN	DOWN	Normal	DOWN	DOWN	DOWN
HSPB6	DOWN	DOWN	DOWN	DOWN	DOWN	DOWN	DOWN	DOWN	DOWN	DOWN	DOWN
ISG20L2	Normal	UP	Normal	NS	NS	UP	UP	Normal	UP	UP	UP
ITIH5	DOWN	DOWN	UP	DOWN	DOWN	DOWN	NS	DOWN	DOWN	DOWN	DOWN
KCNT2	DOWN	DOWN	DOWN	DOWN	DOWN	DOWN	DOWN	DOWN	DOWN	DOWN	DOWN
KIFC2	UP	UP	UP	UP	Normal	UP	UP	UP	UP	UP	UP
LDB2	DOWN	DOWN	DOWN	DOWN	DOWN	DOWN	DOWN	DOWN	DOWN	DOWN	DOWN
MAD2L1	UP	UP	UP	UP	UP	UP	UP	UP	UP	UP	UP
MALL	DOWN	Normal	UP	DOWN	DOWN	DOWN	UP	NS	DOWN	DOWN	DOWN
MCM7	UP	UP	UP	UP	UP	UP	UP	UP	UP	UP	UP
MEX3A	UP	UP	UP	UP	UP	UP	UP	UP	UP	UP	UP
MITF	DOWN	DOWN	UP	DOWN	DOWN	DOWN	DOWN	DOWN	DOWN	DOWN	DOWN
MRVI1	DOWN	DOWN	UP	DOWN	DOWN	DOWN	Normal	DOWN	DOWN	DOWN	DOWN
MTHFD1	Normal	NS	DOWN	Normal	NS	Normal	DOWN	Normal	UP	Normal	Normal
NAA10	UP	UP	Normal	UP	UP	UP	Normal	UP	UP	UP	UP
NALCN	DOWN	DOWN	UP	DOWN	DOWN	DOWN	DOWN	Normal	DOWN	Normal	DOWN
NCAPH	UP	UP	UP	UP	UP	UP	UP	UP	UP	UP	UP
NPR1	DOWN	DOWN	DOWN	DOWN	DOWN	DOWN	DOWN	DOWN	DOWN	DOWN	DOWN
NTRK3	DOWN	DOWN	DOWN	DOWN	DOWN	DOWN	DOWN	DOWN	DOWN	DOWN	DOWN
PDE2A	DOWN	DOWN	DOWN	DOWN	DOWN	DOWN	DOWN	DOWN	DOWN	DOWN	DOWN
PGR	DOWN	UP	DOWN	DOWN	DOWN	DOWN	DOWN	DOWN	DOWN	DOWN	DOWN
PKMYT1	UP	UP	UP	UP	UP	UP	UP	UP	UP	UP	UP
PPIL1	UP	UP	Normal	UP	Normal	NS	UP	UP	UP	UP	UP
PTGDR	DOWN	DOWN	DOWN	DOWN	DOWN	Normal	DOWN	DOWN	DOWN	DOWN	DOWN
RBMS3	DOWN	DOWN	DOWN	DOWN	DOWN	DOWN	DOWN	DOWN	DOWN	DOWN	DOWN
RUNX1T1	DOWN	DOWN	DOWN	DOWN	DOWN	DOWN	DOWN	DOWN	DOWN	DOWN	DOWN
SCARA5	DOWN	DOWN	DOWN	DOWN	DOWN	DOWN	DOWN	DOWN	DOWN	DOWN	DOWN
SETD2	DOWN	NS	DOWN	DOWN	DOWN	DOWN	Normal	Normal	DOWN	Normal	Normal
SGCD	DOWN	NS	NS	DOWN	DOWN	DOWN	DOWN	DOWN	DOWN	DOWN	DOWN

SLBP	UP	UP	NS	UP	UP	Normal	Normal	UP	UP	UP	UP
SLIT2	DOWN	DOWN	UP	DOWN	DOWN	DOWN	DOWN	DOWN	DOWN	DOWN	DOWN
SNRPD1	NS	UP	UP	UP	UP	NS	Normal	UP	UP	UP	UP
SYNE1	DOWN	DOWN	DOWN	DOWN	DOWN	DOWN	DOWN	DOWN	DOWN	DOWN	DOWN
TDRD10	DOWN	DOWN	DOWN	DOWN	DOWN	Normal	Normal	DOWN	DOWN	NS	DOWN
TNXB	DOWN	DOWN	DOWN	DOWN	DOWN	DOWN	DOWN	DOWN	DOWN	NS	DOWN
UBE2C	UP	UP	UP	UP	UP	UP	UP	UP	UP	UP	UP
WDTC1	DOWN	Normal	DOWN	DOWN	DOWN	DOWN	DOWN	Normal	DOWN	DOWN	DOWN
YDJC	UP	UP	NS	UP	UP	Normal	Normal	UP	UP	UP	UP
ZBTB16	DOWN	DOWN	DOWN	DOWN	DOWN	DOWN	Normal	DOWN	DOWN	UP	DOWN
ZNF91	NS	Normal	NS	DOWN	DOWN	DOWN	NS	Normal	Normal	Normal	DOWN

UP —— Up regulated, Down —— Down regulated, NS —— Not significant, Normal —— The gene is neither up regulated nor down regulated.

Table S3. The cancer-specific prognostic markers (HR>1) for 12 cancers with $p<0.05$.

Cancer	Prognostic markers	
	Methylation	Expression
BLCA	ACAN, GIMAP1, CNTN4, HSPB6	RBMS3, HSPB6, FLNC, SLIT2, SGCD, RUNX1T1, MTHFD1, GYPC, PPIL1
BRCA	ACAN, AURKB,	ACAN
CHOL	SCARA5	/
COAD	MEX3A	NALCN, HSPB6, CPEB1, MEX3A
ESCA	/	ZNF91
HNSC	NPR1, ATP1A2, PKMYT1, CDCA8, GIMAP1, TNXB	PPIL1, FLNC, NALCN, CDKN3, BOP1
LIHC	ARHGAP11A, SCARA5	MEX3A, DBF4, CDCA8, BIRC5, ARHGAP11A, CEP55, UBE2C, MAD2L1, AURKB, NCAPH, ISG20L2, CDKN3, SNRPD1, MCM7, AURKA, PPIL1, PKMYT1, SLBP, ACAN, FLNC
LUAD	ZBTB16, AURKB, SETD2, SLIT2	ARHGAP11A, CDKN3, MAD2L1, FLNC, AURKA, PKMYT1, AURKB, NCAPH, MTHFD1, CEP55, CDCA8, UBE2C, SLBP, DBF4, ACAN, CDC37
LUSC	/	PDE2A, GFRA2, ZBTB16, FILIP1, FLNC, ATP1A2, GYPC, SCARA5
PAAD	SNRPD1, NTRK3, ACAN	ARHGAP11A, CEP55, NCAPH, BIRC5, AURKA, UBE2C, CDKN3, AURKB, MTHFD1, MITF, MAD2L1, CDCA8, SNRPD1, SLBP, PKMYT1, DBF4, HBP1
PRAD	NTRK3, SLIT2	DBF4, AURKA, CDCA8, CENPJ, SLBP
READ	NPR1, MCM7, UBE2C	HSPB6, TNXB, TDRD10, PDE2A, GYPC, MITF, RBMS3, PGR, CPEB1, FILIP1, NTRK3

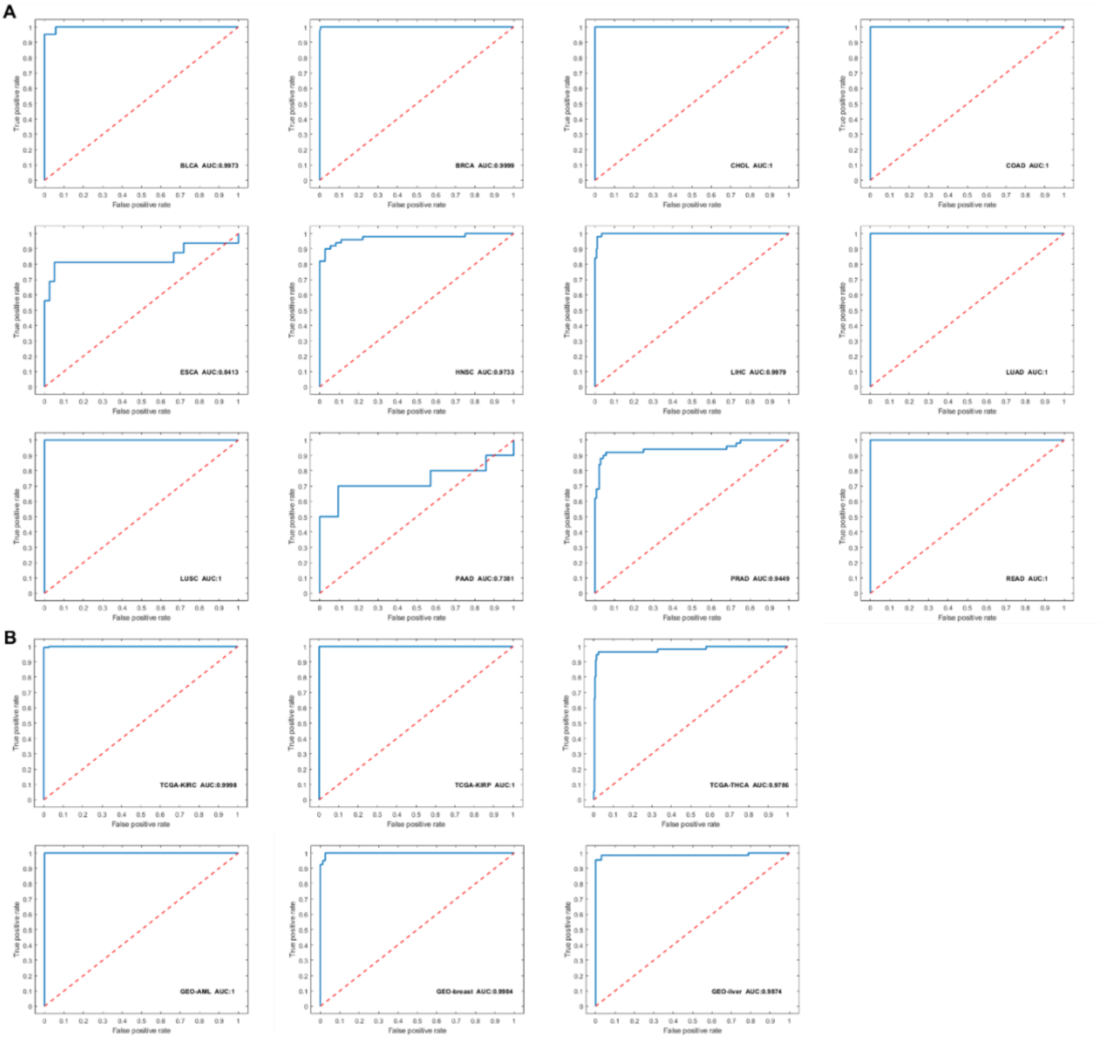

Fig.S1. Receiver operating characteristic (ROC) curve and the area under ROC curve (AUC) of SVM diagnostic model. (A) Training data set. (B) Independent validation set.

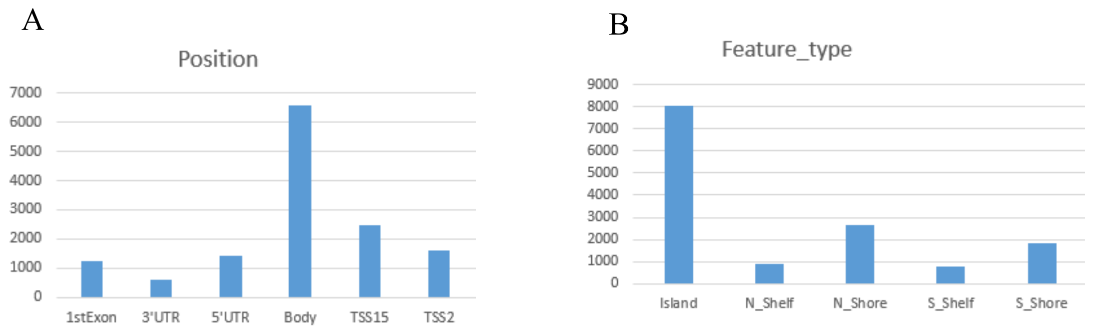

Fig.S2. Distribution of 22,000 potential loci.

Reference

1. Barker M, Rayens W. Partial least squares for discrimination. *J Chemometr.* 2003;17(3):166-173.
2. Wold S, Ruhe A, Wold H, Dunn W. The Collinearity Problem in Linear Regression. The Partial Least Squares (PLS) Approach to Generalized Inverses. *SIAM J SCI COMPUT.* 1984;5(3):735-743.
3. Yang XF, Gao L, Zhang SH. Comparative pan-cancer DNA methylation analysis reveals cancer common and specific patterns. *Briefings in Bioinformatics.* 2017;18(5):761-773.
4. He QE, Tong YF, Ye Z, et al. A multiple genomic data fused SF2 prediction model, signature identification, and gene regulatory network inference for personalized radiotherapy. *Technol Cancer Res T.* 2020;19.
5. Ashburner M, Ball CA, Blake JA. Gene Ontology: tool for the unification of biology. *Nat Genet.* 2000;25(1):25-29.
6. Kanehisa M, Goto S. KEGG: kyoto encyclopedia of genes and genomes. *Nucleic Acids Res.* 2000;28(1):27-30.
7. Yu GC, Wang LG, Han YY, He QY. clusterProfiler: an R package for comparing biological themes among gene clusters. *Omics.* 2012;16(5):284-287.
